# *Pitx3* deficiency promotes age-dependent alterations in striatal medium spiny neurons

**DOI:** 10.3389/fnagi.2022.960479

**Published:** 2022-09-07

**Authors:** Xi Chen, Zhaofei Yang, Yaping Shao, Kunhyok Kim, Yuanyuan Wang, Ying Wang, Haifeng Wu, Xiaolan Xu, Weidong Le

**Affiliations:** ^1^Institute of Neurology, Sichuan Provincial People’s Hospital, University of Electronic Science and Technology of China, Chengdu, China; ^2^Sichuan Translational Medicine Research Hospital, Chinese Academy of Sciences, Chengdu, China; ^3^Liaoning Provincial Key Laboratory for Research on the Pathogenic Mechanisms of Neurological Diseases, The First Affiliated Hospital, Dalian Medical University, Dalian, China

**Keywords:** medium spiny neurons, neuronal morphology, DNA methylation, aging, Parkinson’s disease

## Abstract

**Background:**

The classical motor symptoms of Parkinson’s disease (PD) are tightly linked to the gradual loss of dopamine within the striatum. Concomitantly, medium spiny neurons (MSNs) also experience morphological changes, such as reduced dendritic complexity and spine density, which may be potentially associated with motor dysfunction as well. Thus, MSNs may serve as the emerging targets for PD therapy besides the midbrain dopaminergic neurons.

**Results:**

To comprehensively examine pathological alterations of MSNs longitudinally, we established a *TH*^*Cre/*^*Pitx3*^*fl*/*fl*^ (*Pitx3^cKO^*) mouse model that developed canonical PD features, including a significant loss of SNc DAergic neurons and motor deficits. During aging, the targeted neurotransmitter, MSNs morphology and DNA methylation profile were significantly altered upon *Pitx3* deficiency. Specifically, dopamine, GABA and glutamate decreased in the model at the early stage. While nuclear, soma and dendritic atrophy, as well as nuclear invaginations increased in the aged MSNs of *Pitx3^cko^* mice. Furthermore, more nuclear DNA damages were characterized in MSNs during aging, and *Pitx3* deficiency aggravated this phenomenon, together with alterations of DNA methylation profiling associated with lipoprotein and nucleus pathway at the late stage.

**Conclusion:**

The early perturbations of the neurotransmitters within MSNs may potentially contribute to the alterations of metabolism, morphology and epigenetics within the striatum at the late stage, which may provide new perspectives on the diagnosis and pathogenesis of PD.

## Introduction

The striatum is the largest integrative component of the basal ganglia and plays an essential role in modulating complex behaviors, such as facilitation or inhibition of actions and reward learning (Du and Graves, 2019; Prager and Plotkin, 2019). It receives the glutamatergic afferents from the cerebral cortex and thalamus as well as DAergic afferents from the SNc. These massive neurochemical inputs from corticostriatal, thalamostriatal, and nigrostriatal projections are largely processed by striatal MSNs, together with the interneurons in a topographically organized manner (Bariselli et al., 2019; Filipovic et al., 2019; Chen et al., 2020). MSNs use γ-aminobutyric acid (GABA) as a neurotransmitter and constitute 90–95% of the striatal neuronal population (He et al., 2021). Despite high homogeneity, MSNs can be divided into two distinct subpopulations based on their output projection pathways and neurochemical content (Gerfen et al., 1990; Gong et al., 2003; He et al., 2021). Meanwhile, the MSNs are the only striatal neurons with dendritic spines that are highly specialized structures of neuronal connectivity for the regulation of synaptic strength (Bolam et al., 2000; Parisiadou et al., 2014).

In PD patients, the striatum undergoes progressive DA depletion (Wang et al., 2021), consequently leading to cardinal motor symptoms, such as resting tremor, bradykinesia, postural instability, and rigidity. Meanwhile, MSNs as the predominant striatal neuron population, also experience the morphologic alterations, i.e., the dendritic alterations have been observed in postmortem studies of PD brains (McNeill et al., 1988; Stephens et al., 2005; Zaja-Milatovic et al., 2005). Previously, the toxic PD animal models showed the reduced dendritic length and spine density of MSNs, resembling the findings identified in PD patients (Day et al., 2006; Villalba et al., 2009; Zhang et al., 2013; Suarez et al., 2014; Toy et al., 2014). However, these observations may be due in part to the toxic effects and independent of the PD-induced pathology. Later on, Suarez et al. (2018) represented that the MSNs in *Pitx3* knockout mice do appear similar morphologic abnormalities to that of toxic PD animal models, excluding the toxic effects and suggesting a close association between PD and MSNs. Pitx3 is a transcription factor mainly expressed in midbrain DAergic neurons and plays an essential role in DAergic neuronal development (Smidt et al., 1997, 2004). Later on, our studies showed that Pitx3 is also involved in maintaining the normal physiological functions in the postnatal DAergic neurons (Wang et al., 2021). During aging, the vulnerability of SNc DAergic neurons increased with an early decline in glial cell line-derived neurotrophic factor (GDNF) and aldehyde dehydrogenase 1a1 (Aldh1a1) levels (Wang et al., 2021). Since the deficiency of *Pitx3* triggered a profound loss of SNc DAergic neurons even at the embryonic stage (Hwang et al., 2003; Filali and Lalonde, 2016), few DAergic innervations project onto the striatum and thereby most MSNs are exposed to little DA throughout life, rather than to the progressive depletion of DA during aging. To overcome this drawback and comprehensively examine the pathological alterations of MSNs longitudinally, we generated a *TH*^Cre^*/Pitx3*^fl/fl^** (*Pitx3^cKO^*), a conditional knockout mouse model, where a progressive reduction of striatal DA occurs in the fully developed MSNs. Meanwhile, the mice showed a significant loss of SNc DAergic neurons and movement abnormalities. Thus, the utilization of the *Pitx3^cKO^* model may offer great potential for systematically examining the neurochemistry, morphology and epigenetics of MSNs during aging. Our preliminary studies revealed that multiple neurotransmitters were deducted first in the young *Pitx3^cKO^* mice. The early disruption of neurochemistry may thereby contribute to the remodeling of MSNs late—reduced dendritic complexity of MSNs, and shrinkage of soma and nuclear size, together with altered epigenetic profiling in *Pitx3^cKO^* mice.

## Materials and methods

### The generation of conditional knockout *Pitx3* mouse model

The heterozygous mice *Pitx3*^Flox/wt^ with C57BL/6J background were generated by ViewSolid Biotech Co., Ltd. (Beijing, China) and the TH-Cre driver mice with C57BL/6J background were generated by Shanghai Model Organisms (Shanghai, China); both mouse lines are available upon request. The RioTag mice were purchased from JAX lab (#011029) (Shigeoka et al., 2016). To achieve the conditional knockout *Pitx3* mouse model in the DA neuronal system, *Pitx3^cKO^* mice were produced by breeding mice carrying an *Cre* recombinase under the *TH* promoter with the homozygous mice *Pitx3*^Flox/Flox^. All experimental mice were maintained under specific-pathogen-free (SPF) conditions (temperature, 22°C ± 2°C; air exchange, per 20 min; 12 h/12 h light–dark cycle) with free access to food and water. Animal care and procedures were carried out in accordance with the Laboratory Animal Care Guidelines approved by the Institutional Animal Care Committee at Dalian Medical University. The protocol was approved by the Institutional Animal Care Committee at Dalian Medical University.

For *Pitx3^Flox/wt^* mice, CRISPR technology was applied to cut the DNA of the intron of the *Pitx3* gene, providing the homologous template donor. The sequences of floxp were inserted at both ends of the specific exons (exon 2 and exon 3) through homologous recombination. When mated with tissue-specific expression Cre mice, the specific exon 2 and exon 3 of *Pitx3* were deleted, thereby achieving the purpose of conditional knockout of the *Pitx3* gene. For *TH-Cre* knock-in mouse driver line, the Cre recombinase was cloned following an IRES sequence. An frt-flanked neomycin selection cassette was added, and the construct was cloned in the 3′ untranslated end of the TH gene (as described by Althini et al., 2003). The coding sequence of TH is not affected, nor are the expression levels, so both the TH and Cre recombinase proteins are produced in Th-expressing cells of this mouse line.

### Behavioral test

The open field test was performed in a quiet testing room. To measure the locomotor activity, mice were placed into an Activity Monitor instrument (25 cm × 25 cm × 30 cm, Med Associates Inc., St. Albans, United States) equipped with computer-controlled photocells. Locomotor activity was automatically recorded for 6 min, and different elements of open field test were calculated by the Med system.

Rotarod motor skill learning test was performed as described previously (Wu et al., 2019). Mice were placed onto a rotating rod with auto acceleration from 0 to 40 rpm in 5 min (Model 755, IITC Life Science). The length of time the mouse stayed on the rotating rod was recorded across 10 trials. Such experiments were performed on six continuous days.

### Immunostaining

Mouse brains were collected at indicated time points. The brains were rapidly isolated and postfixed in ice-cold 4% paraformaldehyde and subsequently dehydrated for 24 h in 15% and 30% sucrose at 4°C, as described previously (Dong et al., 2020). Sections were incubated for 1 h in blocking solution (5% normal goat serum, 0.2% Triton-X 100, and 0.05% NaN_3_ in PBS). The primary antibodies were used as follows: anti-TH (1:1,000, AB152; Millipore, United States), anti-TH (1:2,000, T1299; Sigma-Aldrich, United States), anti-TH (1:1,000, TYH, Aves Labs, United States), anti-NeuN (1:1,000, MAB377; Millipore, United States), anti-LaminB1 (1:1,000, 12987-1-AP; Proteintech, United States), anti-Darpp32 (1:1,000, 2306; CST, United States), anti-phospho-Histone H2AX (1:1,000, 2577; CST, United States), anti-GFP (1:1,000, ab6662; Abcam, United States), anti-nuclear pore complex (1:1,000, ab24609; Abcam, United States), anti-TOM20 (1:1,000, 42406; CST, United States), anti-Ctip2 (1:500, ab18465; Abcam, United States), anti-HA (1:500, ab9110; Abcam, United States) and anti-Pitx3 (provided by Dr. Marten P. Smidt’s lab at the University of Amsterdam, Netherlands). The section images were visualized and photographed directly with a confocal microscope (A1 confocal, Nikon Instruments [Shanghai] Co., Ltd.) and a DP80 CCD brightfield microscope (Olympus, Japan). The outlines of the SNc and VTA were delimited according to anatomical landmarks (Fu et al., 2012).

### Image analysis

For neuron counting, a series of coronal sections (40 μm per section, every third section from Bregma –2.70 to –3.88 mm) were selected and stained with anti-TH and anti-NeuN antibodies for quantification. Usually, 10–11 sections were collected per animal. The entire midbrain regions were scanned under a 10X objective (A1 confocal, Nikon Instruments [Shanghai] Co., Ltd.). We multiplied the total calculated from 10 to 11 sections by 3 to obtain the final number of TH^+^ or NeuN^+^ neurons (Dong et al., 2020; Wang et al., 2021). The IFC intensity of the striatum was analyzed using ImageJ software. Typically, the data were collected from 2 to 3 slices per animal.

### Stereotaxic viral injection

The stereotaxic AAV injections (AAV-hSyn1-eGFP, GeneCopoeia) were conducted on 6- and 12-month-old *Pitx3^cWT^* and *Pitx3^cKO^* mice. Before surgery, mice were deeply anesthetized by intraperitoneal injection of ketamine (100 mg/kg)/xylazine (10 mg/kg) solution. To achieve sparse labeling, 1.1 × 10^12^ viral particles with a total volume of 500 nl were injected into dorsal striatum (coordinates used, AP: 0.98 mm, ML: ± 2.2 mm from bregma, DV: –3.0 mm from exposed dura mater). Virus solution was injected at an infusion rate of 100 nl/min and the needle was withdrawn 10 min after the end of injection. Following virus injection, the scalp was sutured, and the mice were returned to their home cages. The virus-injected mice were used for experiment at least 4 weeks after the virus infusion.

### Stereology for neuronal tracing

Based on the previous study (Lu and Yang, 2017), the AAV-infused mouse brains were sectioned at 60 μm of thickness. The sections were stained with GFP antibody (1:1000, ab6662; Abcam, Cambridge, UK) and Ctip2 antibody (1:500, ab18465, Abcam, Cambridge, UK). Afterward, the stained sections were imaged using a laser scanning confocal microscope (A1 confocal, Nikon Instruments [Shanghai]Co., Ltd.) under 40× objective lens. The MSNs were identified based on the positive staining of Ctip2. Neuronal structure reconstruction with neuTube (Feng et al., 2015) and Sholl analysis were performed with ImageJ (Longair et al., 2011).

### Neurotransmitter identification

First, we added 400 μl solution 1 (methanol/water, 1:1, v/v, with 0.1% formic acid) with succinic acid as interior label into the striatum tissue, and the mixture were homogenized. The homogenate was ultrasounded in the ice bath for 10 min, and then was incubated in the ice bath (–20°C) for 30 min. The samples were centrifuged at 12,000 rpm × 10 min under 4°C and 300 μl supernatant was preserved. The pellet was incubated with 200 μl solution 1, vortex for 30 s, and then was ultrasounded in the ice bath for 5 min. The samples were centrifuged at 12,000 rpm × 10 min under 4°C and 200 μl supernatant was preserved. We combined 200 μl supernatant with 300 μl preserved supernatant above and obtained 500 μl supernatant totally. All these 500 μl supernatant was transferred into a glass vial to conduct vacuum-drying. After dissolving and centrifuging, the obtained supernatant was used to identify dopamine, GABA and glutamate using LC-MS system.

### MethylRAD sequencing and DNA methylation data analysis

Genomic DNA was extracted from striatum tissues per mouse at indicated timepoints (QIAamp DNA Micro kit Qiagen, German). MethylRAD library preparation and sequencing were conducted according to the protocol described by Wang et al. (2015). PE sequencing was performed on the Illumina HiSeq X-Ten platform. After QC and filtering of the original reads and removal of the sequences with linkers, low-quality sequences (more than five bases with a quality lower than 10), and those with Ns (unidentified bases), the high-quality clean reads containing the methylated CG/CWG sites were mapped to the reference sequence (signatures with CG/CWG sites) of the mouse genome GENCODE V38 by the SOAP program (version 2.21). Sites covered by at least three reads were regarded as reliable DNA methylation sites. Then, the number of methylated sites and the depth of signature coverage of each sample were calculated. The methylation levels of a site (CG/CWG) could be reflected by the sequencing depth of the methylated signature. The unit of the quantitative value of site methylation was RPM (reads per million), which means that the quantitative value of the methylation level of a site was equal to the coverage at that site in number of reads/the number of high-quality reads in the library multiplied by 1,000,000. Furthermore, the distributions of the methylated CG/CWG sites on different elements of the genome, especially on the different regions of genes, were evaluated by SnpEff software (version: 4.1g) (Cingolani et al., 2012) and bed tools software (v2.25.0) (Quinlan and Hall, 2010). Then, the DNA methylation levels of the genes were evaluated by summing the methylation levels of sites that were localized in the gene region. The differential DNA methylation levels of the sites and genes were identified by using the R package DESeq (Anders and Huber, 2010). Finally, the genes with different methylation levels in different samples were further analyzed based on Gene Ontology (GO) enrichment by DAVID and Kyoto Encyclopedia of Genes and Genomes (KEGG)^1^ enrichment.

### Statistics

Graph Pad Prism 8 and R were used for statistical analysis. The data were collected and processed randomly. No statistical methods were used to predetermine sample size, but our sample sizes are similar to those reported in previous publications. The statistical significance was determined using Student’s *t*- test, 2way ANOVA with Sidak’s multiple comparisons test, conditional logistic regression, and multiple *t*-test with Benjamini and Hochberg test.

## Results

### DAergic neuronal degeneration in *Pitx3^cKO^* mice

Our *Pitx3^cKO^* mouse model utilized a *Cre*-mediated recombination system driven by the *TH* promoter, resulting in the removal of the second and third coding exons of *Pitx3* (Supplementary Figure 1). After multiple generations of breeding, homozygous *Pitx3*-floxed mice harboring (*Pitx3^cKO^*) or not harboring (*Pitx3^cWT^*) the *Cre* gene were achieved, and the genotypes were characterized by PCR analysis (Supplementary Figure 1). The expression of Pitx3 was hardly detected in 2-month-old *Pitx3^cKO^* mice by IFC staining (Supplementary Figure 1), indicating the success of *Pitx3* deletion. Unlike *ak* and traditional Pitx3 knockout mice, *Pitx3^cKO^* mice rarely showed eye defects. Additionally, to confirm the TH-driven Cre expressions, we crossed the RiboTag mice with TH-Cre line (Supplementary Figure 2).

**FIGURE 1:**
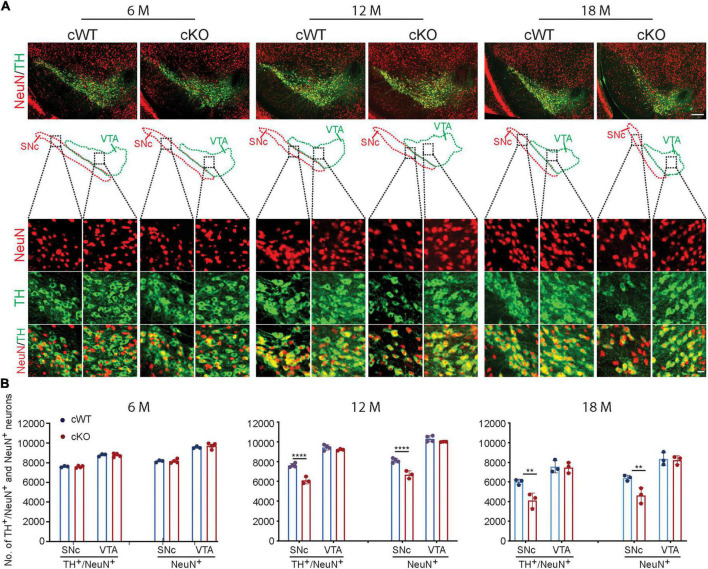
Neurodegeneration in 12- and 18-month-old *Pitx3^cKO^* mice. **(A)** IFC co-staining of TH and NeuN in the ventral midbrain sections from 6-, 12-, and 18-month-old *Pitx3^cWT^* and *Pitx3^cKO^* mice. SNc and VTA were outlined, respectively (scale bar: 200 μm; high-magnification, 20 μm). **(B)** Quantification of TH^+^/NeuN^+^ and NeuN^+^ neurons in the SNc and VTA from 6-, 12-, and 18-month-old *Pitx3^cWT^* and *Pitx3^cKO^* mice (*N* = 3–4 mice per genotype; all males except for two females in 6-month-old *Pitx3^cKO^* and 12-month-old *Pitx3^cWT^*). 2way ANOVA analysis with Sidak’s multiple comparisons test, ****p < 0.0001 (12 months for TH^+^/NeuN^+^ co-staining), ****p < 0.0001 (12 months for NeuN^+^ staining), **p = 0.0041 (18 months for TH^+^/NeuN^+^ co-staining), **p = 0.0064 (18 months for NeuN^+^ staining).

Also, the number of DAergic neurons (Figure 1) and the dendritic complexity of MSN cells (Supplementary Figure 3) were comparable between *Pitx3^cKO^* and *Pitx3^cWT^* mice at the age of 6 months. These results indicated that the development of retina cells, DAergic neurons and MSNs was not disrupted. However, a significant loss of DAergic neurons was characterized in 12-month-old *Pitx3^cKO^* mice, where about 20% of SNc DAergic neurons died (Figure 1). Moreover, such deficit was exaggerated at 18 months with around 31.5% neuronal loss in *Pitx3^cKO^* mice compared to *Pitx3^cWT^* mice (Figure 1). Interestingly, VTA DAergic neurons were less affected by *Pitx3*-deficiency and remained intact during aging (Figure 1), resembling the neuropathological phenotype observed in *ak* and traditional knockout mice. Also, during aging, there is a natural decline in the number of DAergic neurons of 18-month-old *Pitx3^cWT^* mice compared to 12-month-old ones (Supplementary Figure 4). Taken collectively, these results demonstrated the importance of the *Pitx3* gene in adult neuronal survival (Wang et al., 2021).

### Striatal pathology and movement abnormalities in *Pitx3^cKO^* mice

**FIGURE 2:**
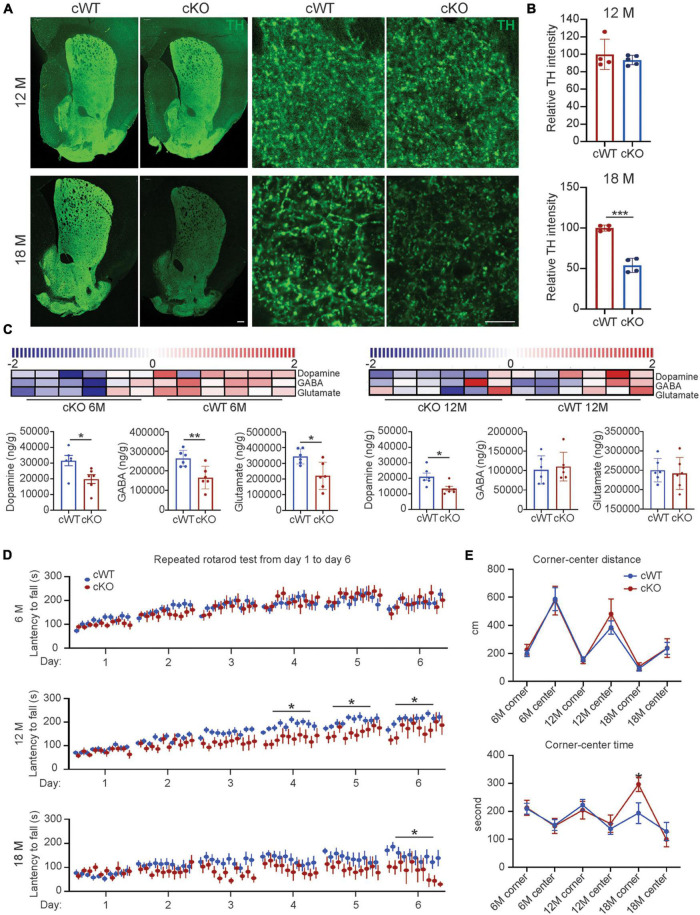
Striatal pathology triggered movement abnormalities in *Pitx3^cKO^* mice. **(A)** IFC staining of TH in the striatal sections from 12- to 18-month-old *Pitx3^cWT^* and *Pitx3^cKO^* mice (scale bar: 200 μm; high-magnification, 10 μm). **(B)** Quantification of relative TH intensity in the striatum from 12- to 18-month-old *Pitx3^cWT^* and *Pitx3^cKO^* mice (*N* = 4 mice per genotype; all males except for two females in 6-month-old *Pitx3^cKO^* and 12-month-old *Pitx3^cWT^*). Unpaired *t*-test, ***p = 0.0006 (18 months). **(C)** Levels of neurotransmitter in 6- (*N* = 6 mice per genotype; all males) and 12-month-old *Pitx3^cKO^* and *Pitx3^cWT^* mice (*N* = 6 mice per genotype; all males). The scaled intensity of three metabolites is relatively depicted according to the color key shown on the above. Red indicates high intensity levels; blue, low intensity levels. Unpaired *t*-test, *p = 0.026 (Dopamine, 6M), **p = 0.0083 (GABA, 6M), *p = 0.012 (Glutamate, 6M), *p = 0.0202 (Dopamine, 12M). **(D)** The latency to fall from rotarod was recorded from *Pitx3^cWT^* and *Pitx3^cKO^* mice at 6 (*N* = 11–13 mice per genotype; all males), 12 (*N* = 11–15 per genotype; all males) and 18 months of age (*N* = 9–11 mice per genotype; all males). 2way ANOVA analysis with Sidak’s multiple comparisons test at 12 and 18 months, *p = 0.0171 (day 4, 12 months), *p = 0.0202 (day 5, 12 months), *p = 0.0376 (day 6, 12 months), *p = 0.0326 (day 6, 18 months). **(E)** Center-corner preference analyses for *Pitx3^cWT^* and *Pitx3^cKO^* mice at 6 (*N* = 11–13 mice per genotype; all males), 12 (*N* = 12–14 per genotype; all males), and 18 (*N* = 9–10 mice per genotype; all males) months of age. 2way ANOVA analysis with Sidak’s multiple comparisons test, *p = 0.05 (time in corner at 18 months).

Besides neuronal loss, our findings further elucidated that the striatal TH expression levels were decreased by 46% in 18-month-old *Pitx3^cKO^* mice (Figures 2A,B), and the reduction of striatal DA levels in *Pitx3^cKO^* mice was identified as early as 6 months of age (Figure 2C). Specifically, a 36% reduction of DA levels was identified in 12-month-old *Pitx3^cKO^* mice, compared to the age-matched *Pitx3^cWT^* mice (Figure 2C). Besides dopamine, we also analyzed the contents of GABA and glutamate within the striatum of mice at the age of 6 and 12 months, respectively. GABA as the primary neurotransmitter of MSNs was substantially decreased in 6-month-old *Pitx3^cKO^*, at which point glutamate also showed a significant reduction in the model (Figure 2C), but there were no significant changes in these two neurotransmitters between the two genotypes at the late stage, suggesting that the striatal GABA and glutamate displayed age-dependent alterations in our model.

The remarkable striatal pathology may contribute to motor behavioral abnormalities (Valentin et al., 2016). We applied a well-adopted repeated accelerating rotarod test (Yin et al., 2009) to evaluate motor skill learning of mice. 6-month-old *Pitx3^cKO^* mice performed equally well with age-matched *Pitx3^cWT^* mice during the 6-day trials, while 12-month-old *Pitx3^cKO^* mice showed markedly fewer improvements after the first 3 days’ training (Figure 2D). Surprisingly, a severely disrupted motor learning phenotype was observed in 18-month-old *Pitx3^cWT^* mice, and *Pitx3*-deficiency can further affect the last day’s training at this advanced stage (Figure 2D). Furthermore, we monitored the voluntary movement of *Pitx3^cKO^* mice in an open-field test at the age of 6, 12, and 18 months. Overall, multiple elements of locomotor activity were strongly age-dependent rather than genotype-dependent, such as distance, rearing and walking speed (Supplementary Figure 5). Additionally, in center-corner behavioral tests, 18-month-old *Pitx3^cKO^* mice prefer to stay in the corner for a longer time, compared to age-matched *Pitx3^cWT^* mice (Figure 2E), suggesting that anxiety levels may be increased. However, the distance traveled in the central zone did not vary between the two genotypes and further investigation may be required, such as an elevated plus-maze test (Rodgers and Dalvi, 1997).

### Morphologic aberrations in medium spiny neurons of *Pitx3^cKO^* mice during aging

**FIGURE 3:**
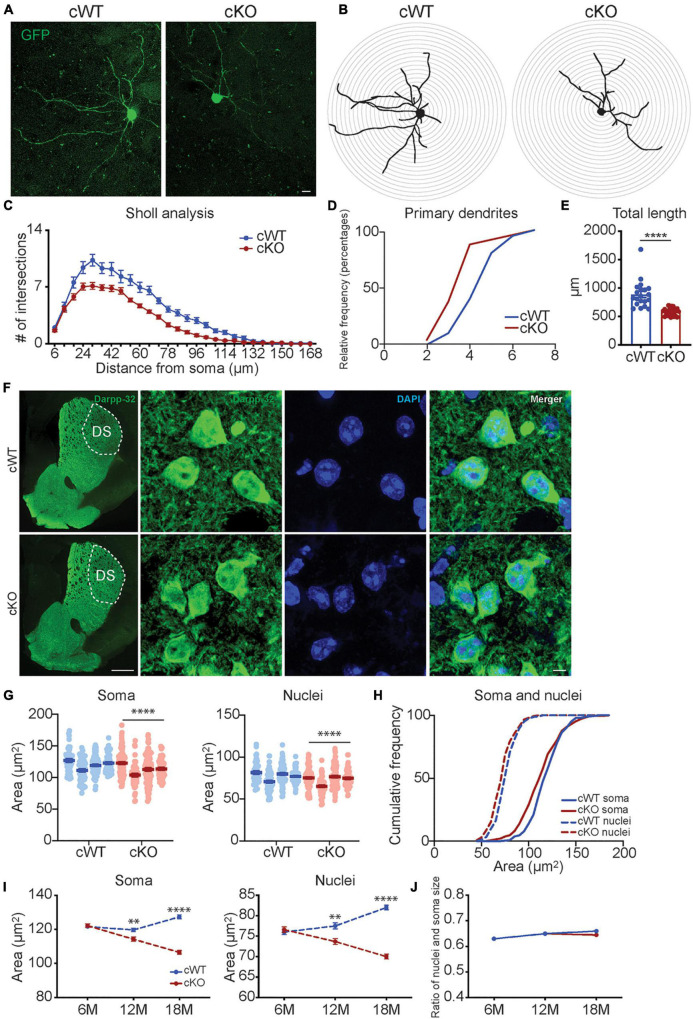
Analyses of neuronal morphology in *Pitx3^cKO^* mice during aging. **(A)** The GFP-labeled individual MSN in 12-month-old *Pitx3^cWT^* and *Pitx3^cKO^* mice (scale bar: 10 μm). **(B,C)** Sholl analysis of dendritic complexity of GFP-labeled MSNs in 12-month-old *Pitx3^cWT^* and *Pitx3^cKO^* mice (*N* = 3 mice per genotype; 5–8 neurons per mouse were counted). Benjamin- Hochberg multiple comparison test of dendritic complexity at 18, 24, 30, 36, 42, 54, 60, 66, 72, 78, 84, 90, 96, 102, 108, and 114 μm from soma, q ≤ 0.05. **(D)** Analyses of primary dendrites (*N* = 3 mice per genotype; 5–8 neurons per mouse were counted; all males). **(E)** Dendritic length of GFP-labeled MSNs in 12-month-old *Pitx3^cWT^* and *Pitx3^cKO^* mice (*N* = 3 mice per genotype; 5–8 neurons per mouse were counted) unpaired *t*-test, ^****^p < 0.0001. **(F)** Co-staining of Darpp32 and DAPI in MSNs of 12-month-old *Pitx3^cWT^* and *Pitx3^cKO^* mice (scale bar: 500 μm; high-magnification, 5 μm). **(G)** The soma and nucleus size of MSNs in 12-month-old *Pitx3^cWT^* and *Pitx3^cKO^* mice (*N* = 4 mice per genotype; all males). Conditional logistic regression test, ^****^p < 0.0001. **(H)** Cumulative frequency of the soma and nuclear size distribution in MSNs of 12-month-old *Pitx3^cWT^* and *Pitx3^cKO^* mice. **(I)** The soma and nucleus size of MSNs in *Pitx3^cWT^* and *Pitx3^cKO^* mice at 6 (*N* = 4 mice per genotype; all males), 12 (*N* = 4 mice per genotype; all males) and 18 months (*N* = 3 mice per genotype; all males) of age. 2way ANOVA analysis with Sidak’s multiple comparisons test, ^**^p = 0.0075 (soma, 12 months), ^****^p < 0.0001 (soma, 18 months), ^**^p = 0.0022 (nuclei, 12 months), ^****^p < 0.0001 (nuclei, 18 months). **(J)** The nuclear size and soma size ratio (N/C ratio) of MSNs in *Pitx3^cWT^* and *Pitx3^cKO^* mice. DS, dorsal striatum.

To examine individual MSN morphology, we stereotactically injected AAV1 vectors into the striatum of 6- and 12-month-old *Pitx3^cWT^* and *Pitx3^cKO^* mice, which express a green fluorescent protein (GFP) under the control of synapsin1 promoter. Due to the low viral titer managed, only a few MSNs with GFP signals could be identified in each hemisphere. We subsequently analyze the dendritic complexity by using 3D reconstruction of individual MSN dendritic trees (Figure 3A and Supplementary Figure 3). The results revealed that a profound reduction in the cumulative length of all dendrites was observed in the MSNs of 12-month-old *Pitx3^cKO^* mice compared to *Pitx3^cWT^* mice (Figures 3A–E). Additionally, dendritic atrophy was age-dependent, since we did not detect any apparent alterations in MSN dendritic length in *Pitx3^cKO^* mice at the age of 6 months (Supplementary Figure 3).

**FIGURE 4:**
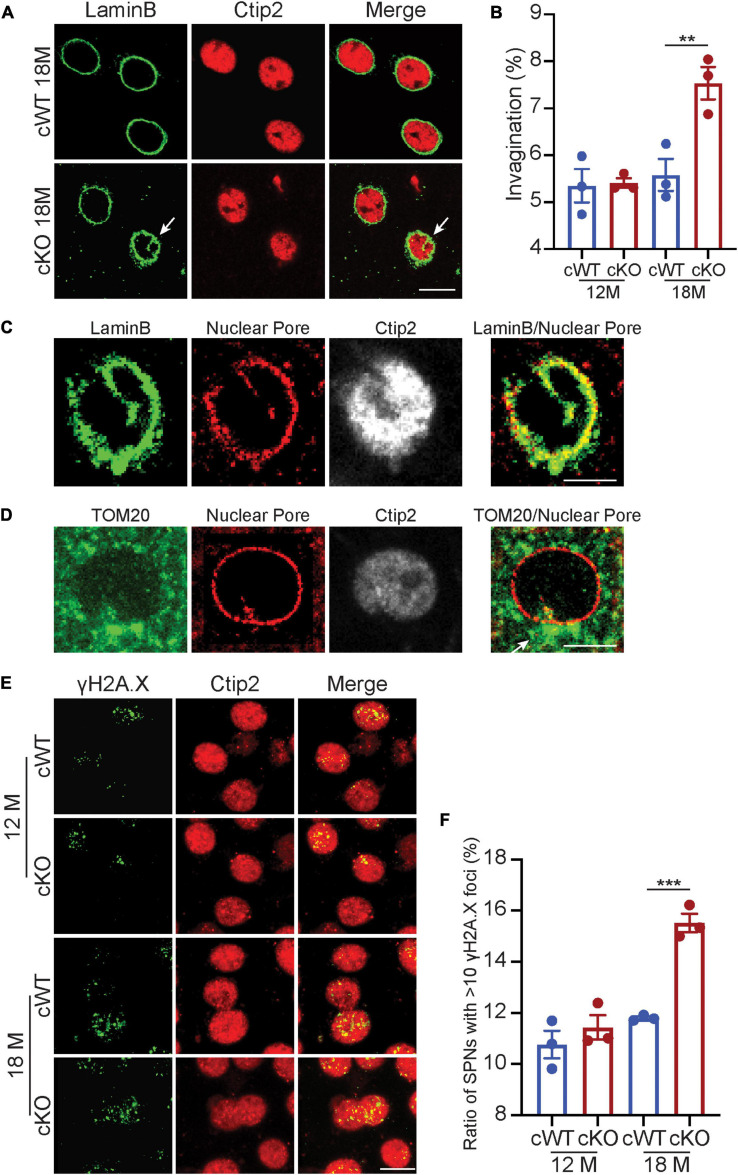
Nuclear invaginations increase accompanied with genomic instability in *Pitx3^cKO^* mice. **(A)** Co-staining of LaminB and Ctip2 in MSNs of 18-month-old *Pitx3^cWT^* and *Pitx3^cKO^* mice (scale bar: 10 μm) white arrow points to nuclear invagination. **(B)** Ratio of MSN nuclei containing ≥ 1 invagination in *Pitx3^cWT^* and *Pitx3^cKO^* mice at 12 (*N* = 3 mice per genotype; all males) and 18 (*N* = 3 mice per genotype; all males) month of age. Unpaired *t*-test, **p = 0.0041. **(C)** Co-staining of Nuclear Pore and LaminB (scale bar: 5 μm). **(D)** Costaining of Nuclear Pore and TOM20 (scale bar: 5 μm). White arrow points to a cluster of mitochondria. **(E)** Co- staining of γH2A.X and Ctip2 in the striatal sections of 12- and 18-month-old *Pitx3^cWT^* and *Pitx3^cKO^* mice (scale bar: 10 μm). **(F)** The ratios of MSNs with 10 or more γH2A.X-positive foci in the nuclei (*N* = 3 mice per genotype). Unpaired *t*-test, ***p = 0.0005.

We further explored the soma morphology of MSNs in 6-, 12-, and 18-month-old *Pitx3^cWT^* and *Pitx3^cKO^* mice and found a marked reduction of the soma and nuclear size in *Pitx3^cKO^* during aging (Figures 3F–I). Our longitudinal data demonstrated that the soma and nuclear size of MSNs were steadily increased in *Pitx3^cWT^* mice from 6 to 18 months of age (Figure 3I). In contrast, the decreased sizes of soma and nucleus in *Pitx3^cKO^* mice were observed from 6 to 18 months of age (Figure 3I). Despite the alterations in the soma and nuclear size, the nucleus to soma ratio (N/C ratio) remained unchanged (Figure 3J). Besides nuclear size, the nuclear shape was altered as well during aging (Figures 4A,B). The increased nuclear invaginations were characterized in 18-month-old *Pitx3^cKO^* mice by immunofluorescent staining, where the nuclear envelope marker LaminB and MSN-specific nuclear marker Ctip2 were used (Figures 4A,B). Additionally, the nuclear pore structures were also identified in the enfolded nuclear envelope (Figure 4C), showing the presence of type II nuclear invagination in the MSNs, i.e., both outer and inner nuclear membranes were enfolded. Furthermore, we identified clusters of mitochondria near the mouth of nuclear invagination within striatal neurons (Figure 4D), which may provide extra ATP and/or calcium buffering capacity to protect against the further deformation of nuclear structures (Drozdz and Vaux, 2017). Nuclear invagination was reported to be closely associated with the expressions of γH2AX, a marker for DNA double-strand breaks and damage (Valdiglesias et al., 2013). We thereby examined the DNA stability in the MSNs of *Pitx3^cWT^* and *Pitx3^cKO^* mice at 12 and 18 months of age. Our results showed a substantial increase in the percentage of MSNs with 10 or more γH2AX-positive foci in the nuclei of 18-month-old *Pitx3^cKO^* mice compared to *Pitx3^cWT^* (Figures 4E,F), corresponding to the observed alterations of nuclear shape. Together, these data suggest that the early neurotransmitters’ disruptions may be involved in regulating nuclear and soma morphology during aging.

### DNA methylation dynamics in *Pitx3^cKO^* mice during aging

To further investigate the role of epigenetics on the whole genome within striatal cells, we used MethylRAD sequencing to analyze the DNA methylation at CG and CWG (W for A or T) sites of *Pitx3^cWT^* and *Pitx3^cKO^* mice’ genome at the age of 12 and 18 months. Overall, the total DNA methylation ratios on CG (methylated CG/total CG sites) were greatly decreased in 18-month-old *Pitx3^cWT^* and *Pitx3^cKO^* (Supplementary Figure 6 and Supplementary Table 1), indicating that aging plays an important role in the global DNA methylation changes. We further examined the distribution patterns of CG methylation sites at the different elements of the genome in all 12 samples (Supplementary Figure 6). The CG methylated sites were concentrated in the introns, followed by the exon and intergenic regions (Supplementary Figure 6). Since CG is more predominant within DNA methylation, we only considered the data from CG sites for the subsequent analyses.

**FIGURE 5:**
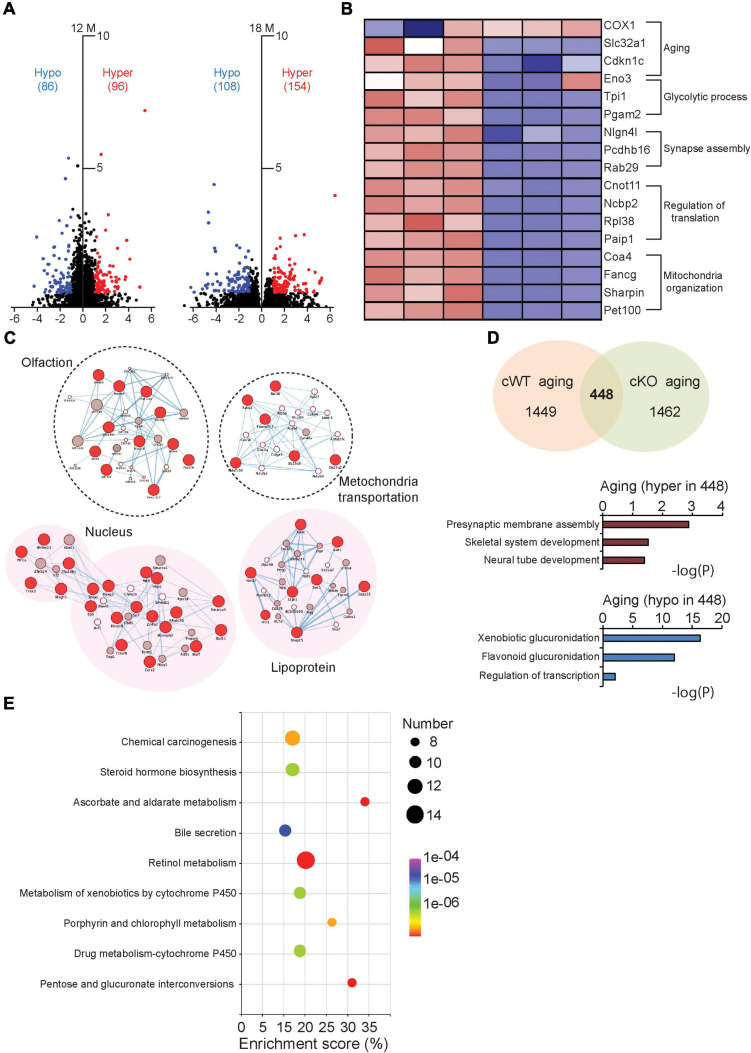
Comparative analysis of the DMGs in *Pitx3^cWT^* and *Pitx3^cKO^* during aging. **(A)** The volcano plots of DNA methylation data collected from the striatum of *Pitx3^cWT^* and *Pitx3^cKO^* mice at 12 (*N* = 3 mice per genotype; all males) and 18 months of age (*N* = 3 mice per genotype; all males). **(B)** Supervised clustering for the DMGs data collected from the 12- and 18-month-old *Pitx3^cWT^* mice. **(C)** Integrated map of GO terms enriched among the DMGs of *Pitx3^cWT^* and *Pitx3^cKO^* mice at 12 (background with white color) and 18 months of age (background with pink color). Red circles represented DMGs identified from our data. **(D,E)** 448 DMGs were identified during aging independent of genotype. They were analyzed with GO terms **(D)** and metabolic pathway **(E)**, respectively.

Besides the global genome examination, the DNA methylation levels of individual genes were also evaluated by summing the methylation levels of sites localized in the gene regions. An analysis of differentially methylated genes (DMGs) was conducted for all the samples. We found 182 DMGs (96 hypermethylated and 86 hypomethylated genes) at 12 months and 262 DMGs (154 hypermethylated and 108 hypomethylated genes) at 18 months, respectively (Figure 5A). Further gene-network studies indicated that the DMGs at 12 months are involved in olfaction and mitochondria transportation pathway, whereas the DMGs at 18 months participate in lipoprotein and nucleus pathway, which may be associated with nuclear morphological changes at this advanced stage (Figure 5C). We further identified the DMGs related to normal aging, characterized from 12- to 18-month-old *Pitx3^cWT^* mice. These genes are involved in multiple cellular process, including aging, glycolytic process, synapse assembly, regulation of translation and mitochondrion organization, consistent with the previous findings (Figure 5B). After crossing these DMGs with the ones characterized from 12- to 18-month-old *Pitx3^cKO^* mice, we totally identified 448 genes, and the alterations of their methylation levels were independent of genotype during aging (Figure 5D). Of them, hypermethylated genes are mainly involved in presynaptic membrane assembly and neural tube development, and hypomethylated genes preferentially participate in xenobiotic glucuronidation and regulation of transcription (Figure 5D). Notably, in the metabolic pathway analysis, retinol metabolism was affected largely, suggesting that retinoic acid within striatal cells could be regulated specifically by the epigenetic way during aging (Figure 5E). Together, these data imply that the DNA methylation modes alters with aging and genotype and may affect multiple cellular process, including retinol metabolism and nuclear pathway, which are potentially associated with the striatal pathology at the advanced stage.

## Discussion

Here we demonstrated that *Pitx3^cKO^* mice showing PD-related features, such as reduced DA and DAergic neuronal degeneration. Meanwhile, besides DA, the homeostasis of GABA and glutamate was impaired at the early stage in the model, potentially contributing to the striatal pathologies at the late stage. Furthermore, we novelly characterized nuclear atrophy and nuclear invagination increase in MSNs during aging, and these aberrant nuclear phenotypes may be associated with epigenetic alterations at the advanced stage.

In PD, the deficit of midbrain DAergic neurons produces the reduction of DA in the basal ganglia (Weintraub et al., 2022). Our *Pitx3^cKO^* mice showed a significant reduction of DA at 6 months, and the deficit aggravated at the late stage. Thus, the model provided great potential for studying the age-dependent striatal pathologies under progressive DA depletion. First, we examined the levels of two main neurotransmitters, GABA and glutamate, since they may be involved in remodeling the plasticity of MSNs, synergistically with DA. Interestingly, GABA and glutamate were both decreased at the early stage of our model, compared to controls. Previously the remarkable alterations of GABAergic neurotransmission within the basal ganglia circuit were reported in PD (Jamwal and Kumar, 2019). Moreover, in MPTP mice, the decreased levels of GABA in the striatum have been characterized, where about 75–80% of SNc were lost (Singh et al., 2017). However, in our studies, the significantly altered GABA levels were only characterized in the young *Pitx3^cKO^* mice, i.e., there were no changes in GABA levels between the two genotypes at the advanced stage, indicating that an adaptive system may respond to restoring the GABA levels in our model during aging. On the other hand, like GABA, the perturbation of glutamate homeostasis also altered in our model in an age-dependent way, i.e., the notably changed glutamate levels in *Pitx3^cKO^* mice were only identified in the early stage, but not in the late stage. However, our glutamate results were inconsistent with the previous studies that the enhancement of glutamate content was associated with the robust MSNs hyperactivity in the PD-related animal models (Calabresi et al., 1993; Singh et al., 2015; Tozzi et al., 2021). Noticeably, these models were majorly administrated with 6-OHDA, MPTP, or α-syn-PFF, rather than genetic models. One of the outstanding features of these models is the occurrence of the severe loss of DAergic neurons, usually reaching 70–80%, whereas, in genetic models, the death rate of DAergic neurons typically reached 40–50% (Konnova and Swanberg, 2018). Thus, the differences in DAergic neuron loss among the animal models may affect the glutamate release in the striatum. Additionally, when determining neurotransmitter levels, we used whole striatum tissue extracts for HPLC analysis, which may compromise the subtle changes occurring at the extracellular/synaptic levels. In the future, the use of microdialysis will be another better choice. Taken together, the neurotransmitter levels were altered at 6 months, while the MSNs of *Pitx3^cKO^* mice remained integrity at that time. During aging, the atrophy of dendritic complexity, soma and nuclei was identified in the MSNs of 12-month-old *Pitx3^cKO^* mice, concomitated with the significant loss of SNc neurons and movement abnormalities. We thereby suggested that the early perturbation of neurotransmitters may progressively trigger the striatal pathologies.

MSNs in our model have shorter dendritic lengths and lower maximal branch order of the dendrites at 12 months. This phenotype is similar to what is observed in patients and the animal models of PD (McNeill et al., 1988; Toy et al., 2014). Unlike *ak* and traditional *Pitx3* knockout mice, MSNs develop and mature with abundant DA afferents in our genetic model. Thus, the observed defects of tree complexity exclude the cause of developmental defects. Besides dendritic shortage, we identified nuclear morphological alterations in MSNs. Irregular shapes of nuclei have been reported in the neurons of PD patients with LRRK2-related G2019S (Liu et al., 2012; Shani et al., 2019), transgenic mice carrying R1441C mutations (Tsika et al., 2014) and LRRK2 knockout mice (Chen et al., 2020). As an extension of these findings, our present studies demonstrated that MSNs showed reduced nuclear size and increased nuclear invagination during aging. The decreased size of the nucleus likely reflects lower biosynthetic activities of DNA repair/synthesis, transcription, and translation in cells (Koda et al., 2006), which potentially contributed to the striatal neuronal dysfunction at the advanced stage. On the other hand, in our mouse model, increased nuclear invagination was characterized at the advanced stage, reflecting that the higher neuronal excitability might occur over there (Chen et al., 2020). Comparably it was reported that the depolarizing current significantly evoked more action potentials in MSNs of *Pitx3* knockout mice (Suarez et al., 2018). Thus, our results re-emphasize that increased neural activity facilitates nuclear invagination formation. Together, not only the dendritic complexity but also the nuclear morphology alters in MSNs upon PD-like stress during aging. Especially, the aberrant nuclear phenotype may bring out severe effects on genomics, and further impact the downstream cellular progresses.

Age-related remodeling of DNA methylation comprises events of both hypo- and hypermethylation (Miranda-Morales et al., 2017; Yang et al., 2022). These epigenetic alterations mediated heritable changes in the gene activity and contributed to genomic instability. Our data showed that increased γH2AX was observed in the MSNs of 18-month-old *Pitx3^cKO^* mice, indicating that DNA damage might be persistent and accumulative during the aging process. To further elucidate the whole-genome epigenetic changes during aging, we performed DNA methylation studies between *Pitx3^cKO^* and *Pitx3^cWT^* mice at 12 and 18 months. Our data showed that the total DNA methylation ratios were greatly decreased in 18-month-old mice compared to 12-month-old ones, consistent with previous studies that DNA hypomethylation occurs globally over time (Miranda-Morales et al., 2017). For methylation onto individual genes, the previous studies reported that the MAPT gene was hypomethylated in the putamen of PD patients’ post-mortem brains (Coupland et al., 2014). Whereas in our longitudinal epigenetic data, many DMGs involved in the nucleus pathway were characterized in *Pitx3^cKO^* mice at the advanced stage, potentially associating with their nuclear morphological alterations. However, what factor contributed to the changes in DNA methylation profile? Recent studies indicated that DA could modify histone H3 glutamine 5 (H3Q5dop) to regulate cocaine-induced transcriptional plasticity in the midbrain (Lepack et al., 2020). The non-neurotransmission roles of neurotransmitters in the epigenetic process have attracted more attention recently. In our model, the progressive reduction of DA and temporally altered neurotransmitters have been identified, thus the perturbation of neurochemicals might affect the epigenetic changes and further remodel the neuronal plasticity. More detailed biochemical and molecular mechanisms of how neurotransmitters regulate neuronal plasticity are also needed in the future.

Previously, we reported another Pitx3 conditional knockout model, *Pitx3*^*fl/fl/*^*DAT*^*CreERT*2^ mice (Wang et al., 2021). Similar to the *DAT* model, our model has progressive DAergic neuron degeneration and DA reduction during aging, greatly different from *ak* and *Pitx3^–/–^* mice that showed about 60% SNc neuron loss and 80% striatal DA reduction as early as the age of 2 months. One of the reasons why our model facilitates the development of DAergic neurons may be that Pitx3 in SNc is expressed earlier than TH during development (Maxwell et al., 2005). Thus, prior to Cre recombination, some Pitx3 proteins are already present and exert the biological function, i.e., a serials of downstream development events have been triggered. Furthermore, Pitx3 as a transcription factor is considered to modulate TH expressions (Cazorla et al., 2000). Therefore, decreased Pitx3 expressions may reduce the efficiency of the TH-driven Cre-loxp system. Taken together, these contributing factors may aid in the development of DAergic neurons in our model. However, whether the development of DAergic neurons is intrinsically affected in our model needs to be further investigated by examining the expression levels of development-related molecules, such as DAT, Vmat2, Aadc, and so on.

## Conclusion

The striatum is the key player in facilitating voluntary movement. In PD, the striatal neurons undergo the progressive depletion of DA, resulting in impaired physiological function and contributing to the motor symptom of PD. To further explore the striatal pathologies during aging, we generated the *Pitx3^cKO^* mice, where a progressive reduction of striatal DA was identified. In this model, the levels of GABA and glutamate decreased at the early stage besides DA. Such early disturbance of neurochemical homeostasis may contribute to the longitudinal plasticity remodeling of neurons, including morphology and movement abnormalities as well as aberrant epigenetic modifications. Our studies may expand the overview of PD treatments and provide a new potential window for therapeutic strategies.

## Data availability statement

The datasets presented in this study can be found in online repositories. The names of the repository/repositories and accession number(s) can be found below: https://submit.ncbi.nlm.nih.gov/subs/bioproject/SUB11723472/overview.

## Ethics statement

This animal study was reviewed and approved by the Institutional Animal Care Committee at Dalian Medical University.

## Author contributions

XC and WL designed the experiments, wrote, and edited the manuscript. ZY contributed to stereotactic injection. XC and YS contributed to metabolic analyses. XC and YiW contributed to imaging experiments and data analysis. KK, YuW, and HW contributed to behavior test and data analysis. XC and XX contributed to sample preparation and data analysis for DNA methylation. All authors read and approved the final manuscript.

## Footnotes

1
http://www.genome.jp/kegg/

## Supplementary material

Supplementary Figure 1 Conditional knockout of *Pitx3* in DA neurons. **(A)** The basic strategy for the generation of *TH*^Cre^*/Pitx3*^fl/fl^** knockout mice. **(B)** PCR detection of *Cre* transgene (upper) and *Pitx3* floxed allele (lower). **(C)** IFC staining for Pitx3 expression in DA neurons was performed using an antibody against Pitx3 (green) together with TH (red) in 2-month-old *Pitx3^cWT^* and *Pitx3^cKO^* mice (scale bar: 100 μm; high-magnification, 5 μm).

Supplementary Figure 2 Specific labeling of DAergic neurons using RiboTag mice (Scale bar: 100 μm).

Supplementary Figure 3 Analyses of dendritic complexity in 6-month-old *Pitx3^cKO^* mice. **(A)** The GFP-labeled individual MSN (scale bar: 10 μm). **(B,C)** Sholl analysis of dendritic complexity of GFP-labeled MSNs (*N* = 3 mice per genotype; 5–8 neurons per mouse were counted; all males). **(D)** Analyses of primary dendrites (*N* = 3 mice per genotype; 5–8 neurons per mouse were counted; all males). **(E)** Dendritic length of GFP-labeled MSNs (*N* = 3 mice per genotype; 5–8 neurons per mouse were counted; all males).

Supplementary Figure 4 TH^+^ neurons in 12 and 18-month-old *Pitx3^cWT^* mice (scale bar: 200 μm).

Supplementary Figure 5 Open field test for *Pitx3^cWT^* and *Pitx3^cKO^* mice at 6 (*N* = 11–13 mice per genotype; all males), 12 (*N* = 12–14 mice per genotype; all males), and 18 months of age (*N* = 9–10 mice per genotype; all males).

Supplementary Figure 6 Overview of the DNA methylation ratio and distribution of CG sites in *Pitx3^cWT^* and *Pitx3^cKO^* mice. **(A)** The genomic DNA methylation ratio at CG and CWG sites in 12- and 18-month-old *Pitx3^cWT^* and *Pitx3^cKO^*. **(B)** The distribution of CG sites on different functional genic components. Upstream, downstream, exon, and intron indicate the regions 2,000 bp upstream of the transcription start site, the regions 2,000 bp downstream of the transcription terminal site, the whole exons of genes, and the whole introns of genes, respectively. 3′UTR and 5′UTR indicate the regions at the 3′ end and 5′ end of a mature transcript that are not translated into a protein. Intergenic indicates the intergenic regions.

Supplementary Table 1 DNA methylation ratios in 12- and 18-month-old *Pitx3^cWT^* and *Pitx3^cKO^* mice.

## Abbreviations

PD: Parkinson’s disease
MSNs: spiny projection neurons
DA: dopamine
TH: tyrosine hydroxylase
SNc: substantia nigra
GABA: γ-aminobutyric acid
*ak*: aphakia
VTA: ventral tegmental area
GFP: green fluorescent protein
DMGs: differentially methylated genes.
