# Supplementary figures and images for: Pitx3 deficiency promotes age-dependent alterations in striatal medium spiny neurons
Source: Front Aging Neurosci. 2022 Sep 7;14:960479. doi: 10.3389/fnagi.2022.960479 (PMC9490232; doi:10.3389/fnagi.2022.960479)

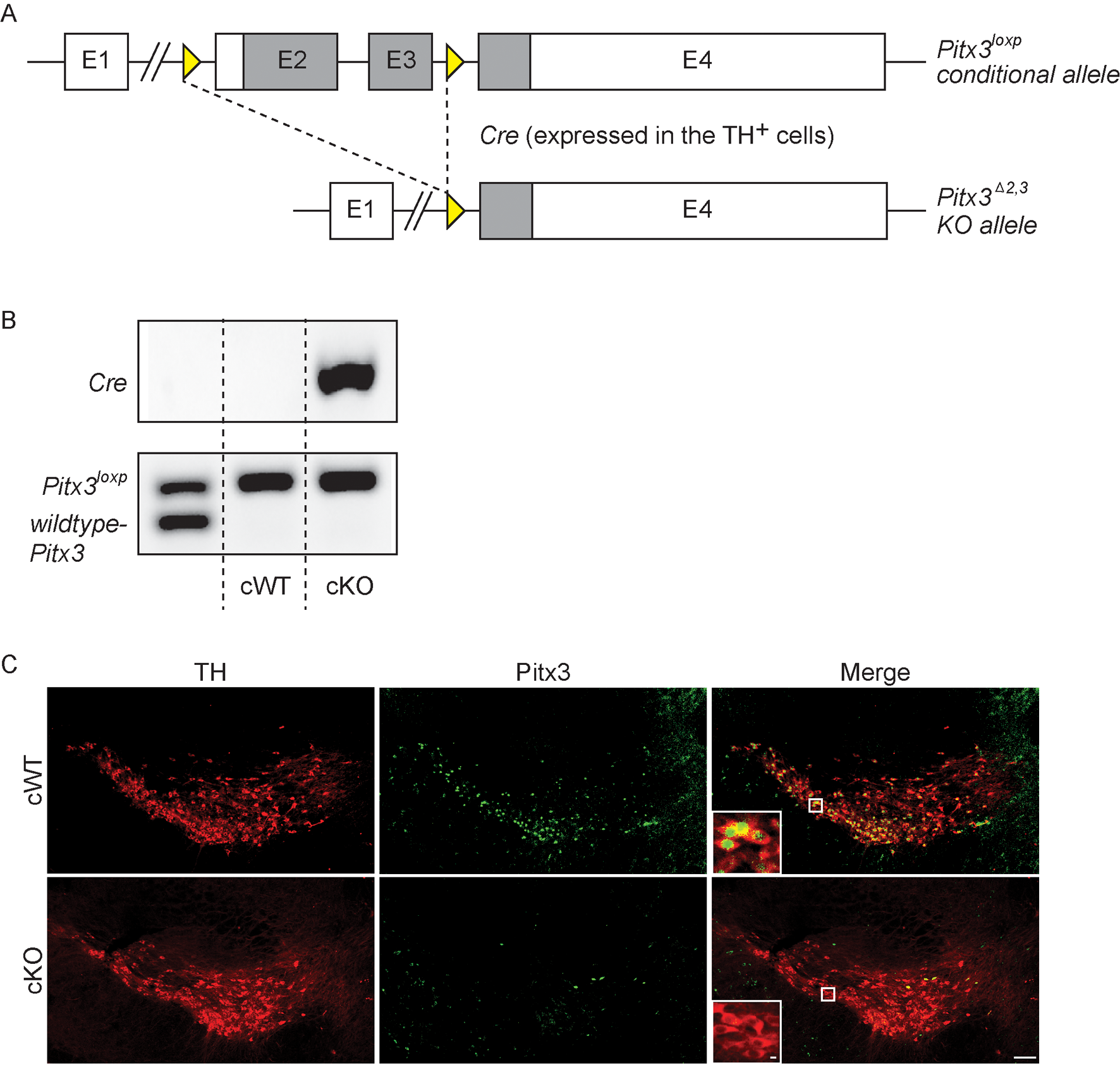

Supplement: Supplementary Figure 1 — Conditional knockout of Pitx3 in DA neurons. (A) The basic strategy for the generation of THCre/Pitx3fl/fl knockout mice. (B) PCR detection of Cre transgene (upper) and Pitx3 floxed allele (lower). (C) IFC staining for Pitx3 expression in DA neurons was performed using an antibody against Pitx3 (green) together with TH (red) in 2-month-old Pitx3cWT and Pitx3cKO mice (scale bar: 100 μm; high-magnification, 5 μm). [file Image_1.TIF]

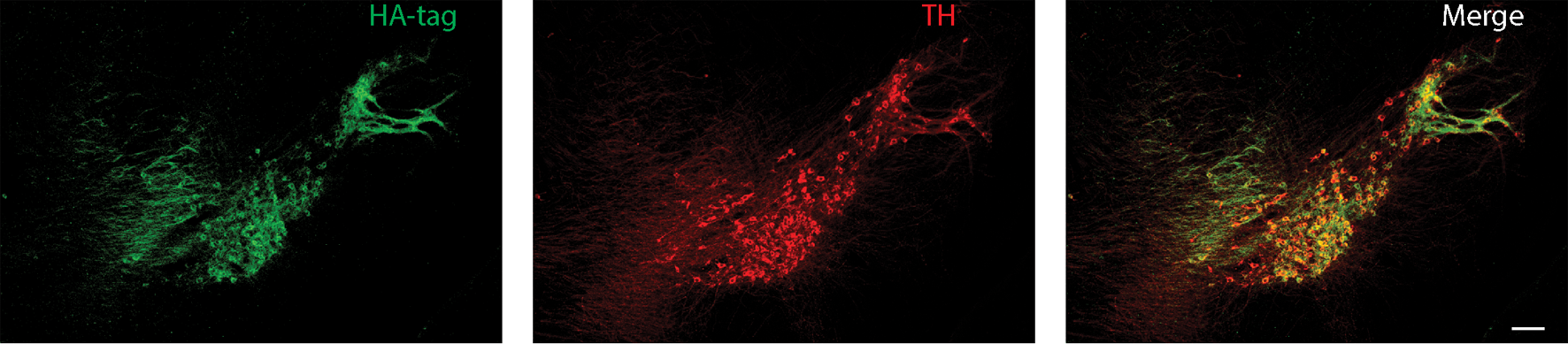

Supplement: Supplementary Figure 2 — Specific labeling of DAergic neurons using RiboTag mice (Scale bar: 100 μm). [file Image_2.TIF]

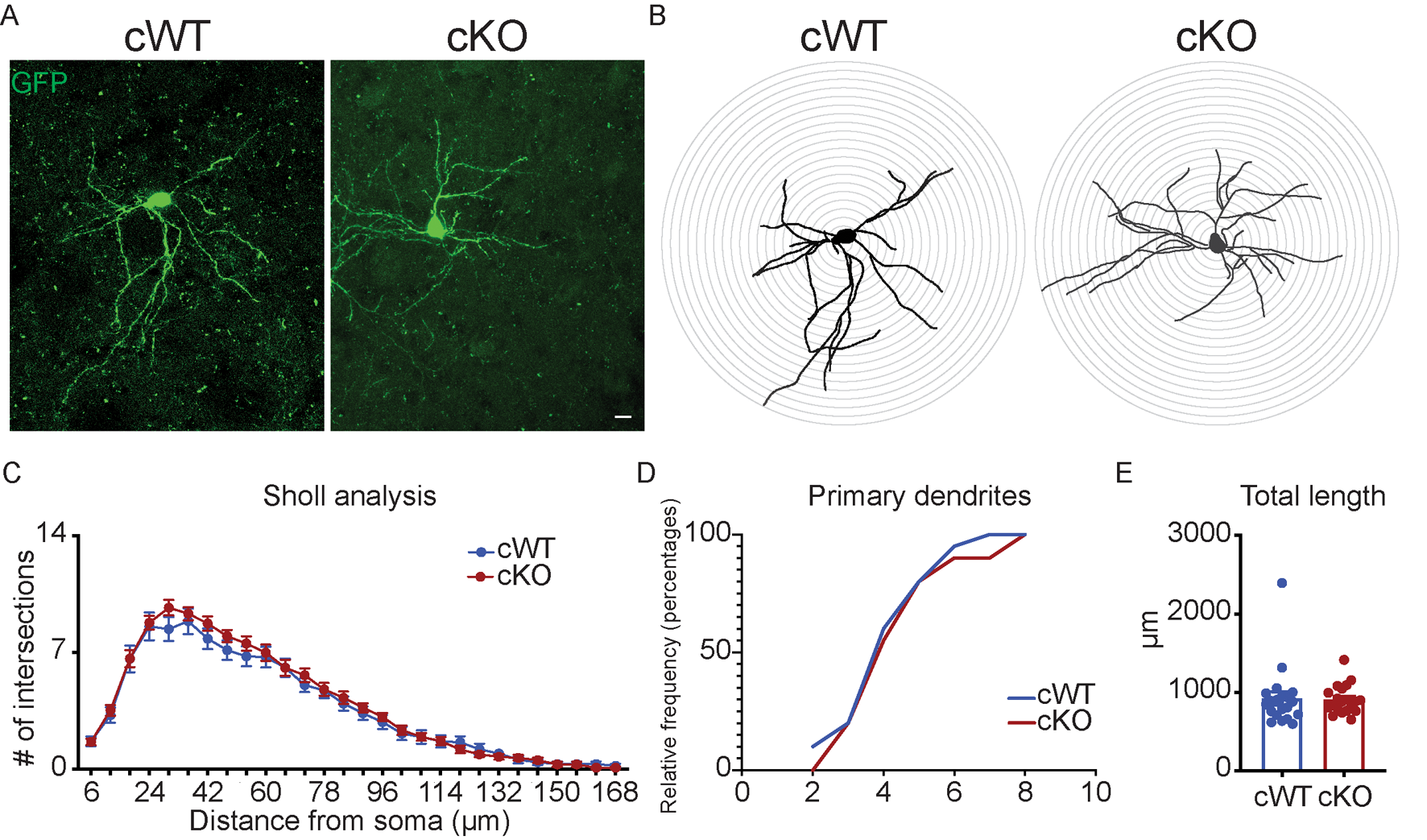

Supplement: Supplementary Figure 3 — Analyses of dendritic complexity in 6-month-old Pitx3cKO mice. (A) The GFP-labeled individual MSN (scale bar: 10 μm). (B,C) Sholl analysis of dendritic complexity of GFP-labeled MSNs (N = 3 mice per genotype; 5–8 neurons per mouse were counted; all males). (D) Analyses of primary dendrites (N = 3 mice per genotype; 5–8 neurons per mouse were counted; all males). (E) Dendritic length of GFP-labeled MSNs (N = 3 mice per genotype; 5–8 neurons per mouse were counted; all males). [file Image_3.TIF]

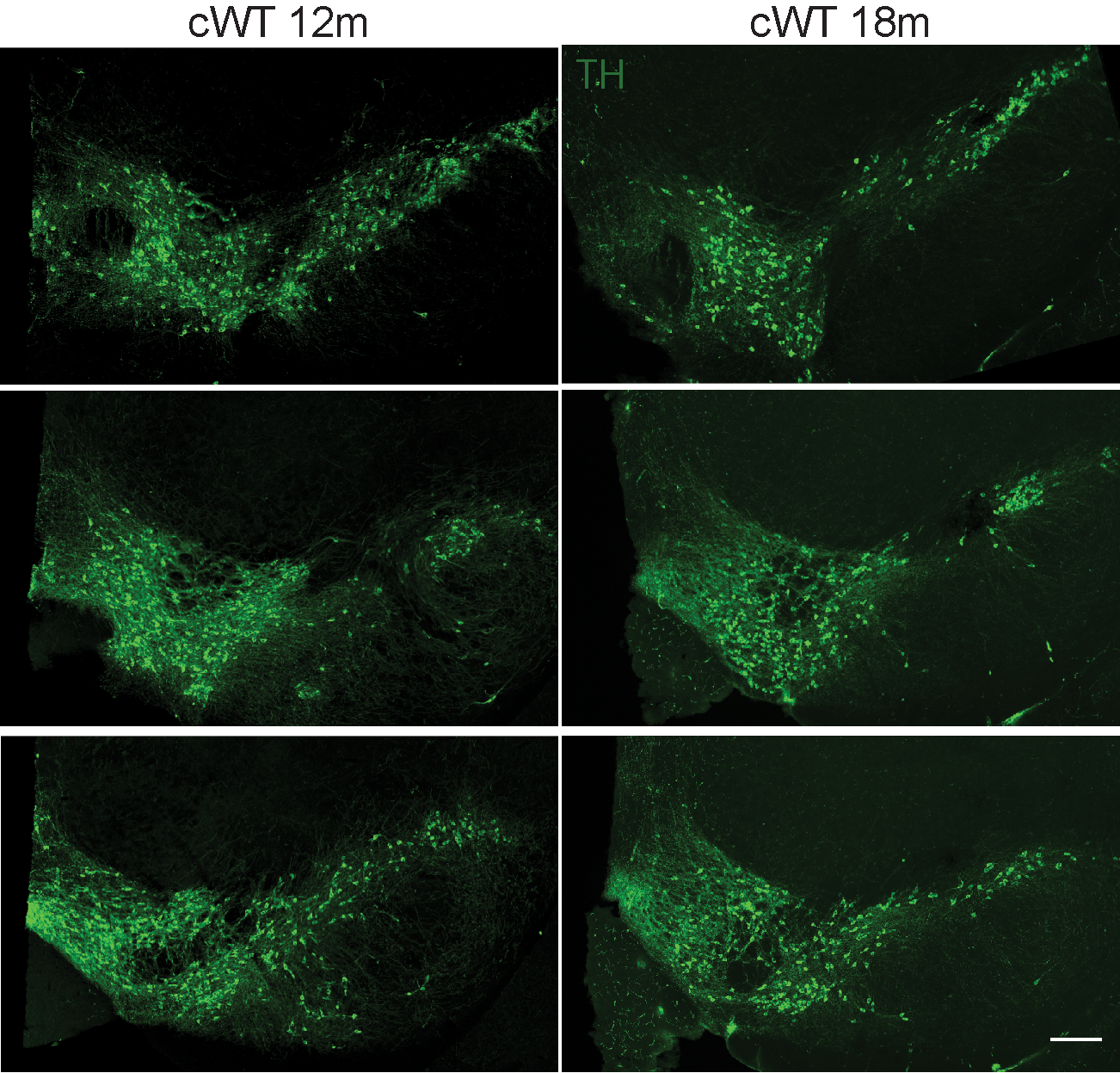

Supplement: Supplementary Figure 4 — TH+ neurons in 12 and 18-month-old Pitx3cWT mice (scale bar: 200 μm). [file Image_4.TIF]

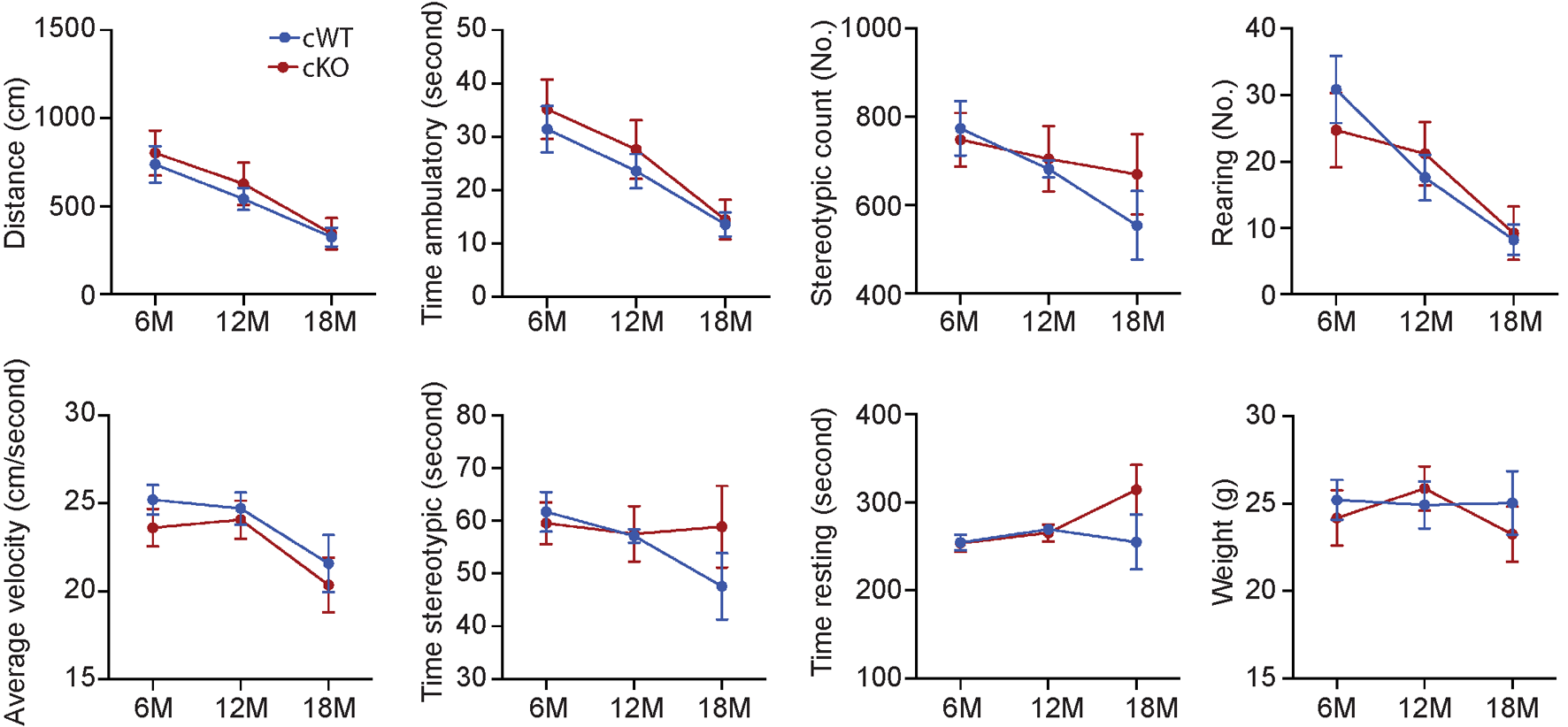

Supplement: Supplementary Figure 5 — Open field test for Pitx3cWT and Pitx3cKO mice at 6 (N = 11–13 mice per genotype; all males), 12 (N = 12–14 mice per genotype; all males), and 18 months of age (N = 9–10 mice per genotype; all males). [file Image_5.TIF]

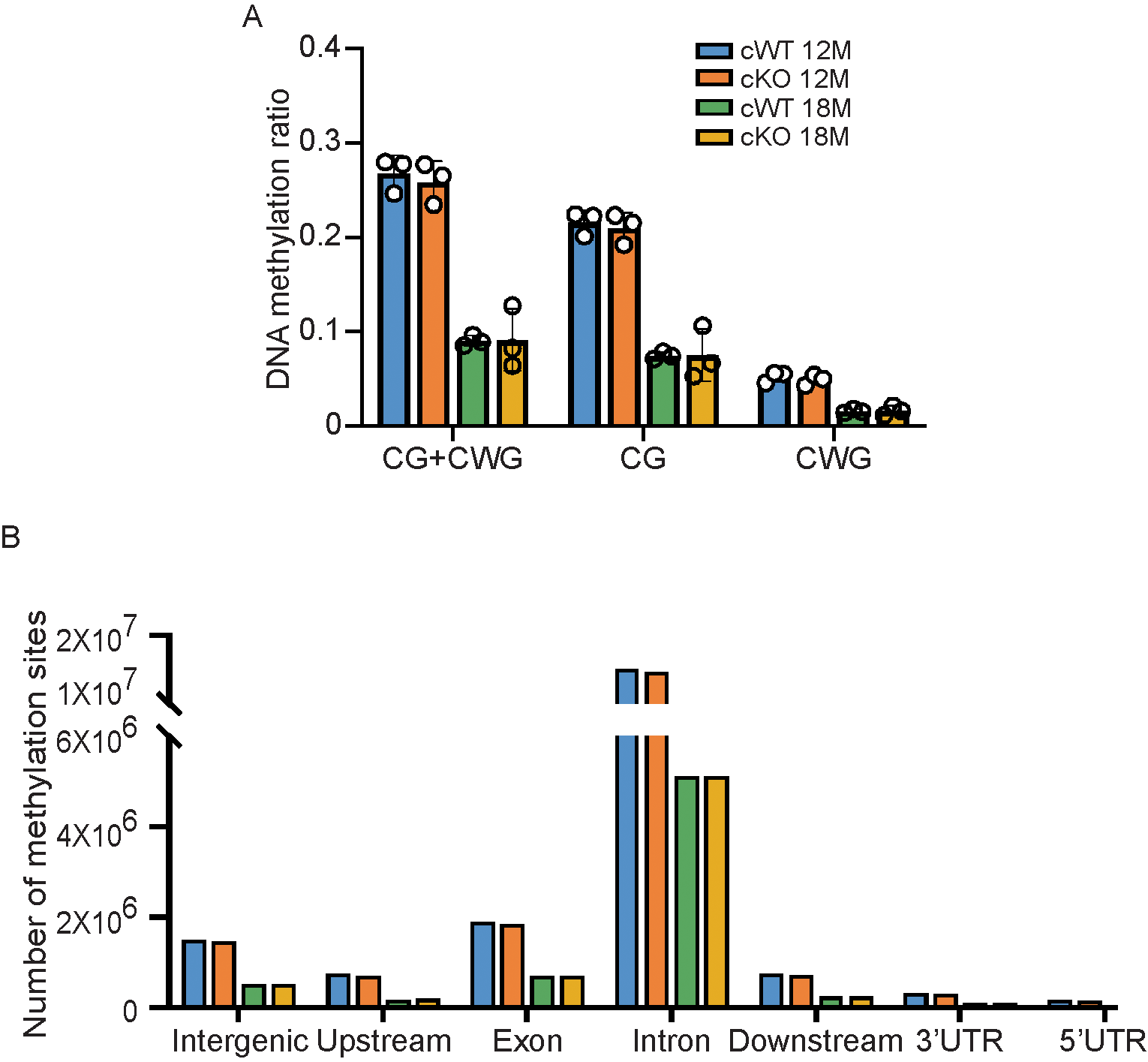

Supplement: Supplementary Figure 6 — Overview of the DNA methylation ratio and distribution of CG sites in Pitx3cWT and Pitx3cKO mice. (A) The genomic DNA methylation ratio at CG and CWG sites in 12- and 18-month-old Pitx3cWT and Pitx3cKO. (B) The distribution of CG sites on different functional genic components. Upstream, downstream, exon, and intron indicate the regions 2,000 bp upstream of the transcription start site, the regions 2,000 bp downstream of the transcription terminal site, the whole exons of genes, and the whole introns of genes, respectively. 3′UTR and 5′UTR indicate the regions at the 3′ end and 5′ end of a mature transcript that are not translated into a protein. Intergenic indicates the intergenic regions. [file Image_6.TIF]
